# Ethical implications of nurse brain drain on undergraduate nursing students

**DOI:** 10.1177/09697330251374392

**Published:** 2025-09-04

**Authors:** Animesh Ghimire, Mamata Sharma Neupane

**Affiliations:** 1 School of Nursing and Midwifery, 2541 Monash University, Clayton, VIC, Australia; 2 Sustainable Prosperity Initiative Nepal, Kathmandu, Nepal; 3 School of Nursing, 475236 Chitwan Medical College, Bharatpur, Nepal; 4 School of Public Health, 475236 Chitwan Medical College, Bharatpur, Nepal

**Keywords:** brain drain, moral distress, Nepal, phenomenology, migration, global health workforce

## Abstract

**Background:**

Global healthcare worker migration, often termed “brain drain,” poses profound ethical challenges for low- and middle-income countries (LMICs) such as Nepal. Although the economic and professional drivers behind nurse migration are relatively well-documented, the ethical implications for nursing students—who witness this dynamic during their formative training—remain insufficiently examined.

**Aim:**

This study investigated how undergraduate nursing students in Nepal perceive and navigate the ethical dimensions of nurse brain drain.

**Research Design:**

A descriptive phenomenological design was employed. Data was collected through semi-structured interviews and analyzed using a thematic approach.

**Participants and Research Context:**

Sixteen third- and fourth-year Bachelor of Science in Nursing students at a tertiary institution in Chitwan, Nepal, were purposively sampled.

**Ethical Considerations:**

The Nepal Health Research Council (NHRC-277/2024) granted ethical approval. Informed consent was obtained from all participants, who were assured of their right to confidentiality and withdrawal rights.

**Results:**

The findings revealed a profound ethical narrative captured in five interconnected themes. Participants are caught in “The Dissonance of Duty,” a core conflict exacerbated by witnessing “The Eroding Ideal” of their profession within a strained system and the subsequent “Ripple Effect of Absent Role Models.” This compels a complex process of “justifications, rationalizations, and lingering doubts regarding migration.” Ultimately, many resolve this tension by “Reimagining Patriotism in a Globalized Profession,” framing their potential departure as a new form of transnational contribution.

**Conclusions:**

Nepalese nursing students are active moral agents, rethinking duty and national allegiance in a context marked by workforce shortages and global opportunity. Addressing these ethical dilemmas demands curricular reforms emphasizing real-world decision-making, transnational mentorship opportunities, and systemic improvements in working conditions. Fostering an environment that inspires rather than compels loyalty is crucial for sustaining Nepal’s healthcare workforce—a lesson of considerable relevance to other LMICs confronting similar migration pressures.

## Introduction

The global migration of healthcare professionals, often termed “brain drain,” presents a significant and complex challenge, particularly for lower- and middle-income countries (LMICs) that experience a disproportionate loss of skilled workers.^1^ This phenomenon is not merely a labor market adjustment; it’s a symptom of deeper systemic inequalities. Nurses from LMICs are often drawn to higher-income countries (HICs) by the promise of improved working conditions, higher salaries, and opportunities for specialized training—factors that collectively contribute to critical workforce shortages in their home countries.^2–5^ This transnational movement carries complex ethical ramifications, both for the sending nations striving to maintain functional healthcare systems and for individual nurses balancing personal gain against broader societal obligations.

## Background

Nepal exemplifies these challenges, experiencing one of the highest rates of nurse emigration in its region.^1,6^ Thousands of Nepali nurses seek employment abroad annually, primarily in countries like the United Kingdom (UK), Australia, the United States (US), and Canada.^7,8^ This exodus is fueled by a confluence of well-documented push and pull factors. Key push factors include challenging working conditions—such as high patient loads, limited resources, and concerns about workplace safety—that drive nurses away from the domestic health sector.^3,9–12^ In parallel, powerful pull factors, including established migration networks and bilateral recruitment agreements designed to facilitate recruitment by HICs, provide facilitated pathways to work abroad.^2,13^ Within Nepal’s historical context, where migration for better opportunities is often viewed as a necessity, this “brain drain” significantly weakens the national healthcare sector and raises critical questions about distributive justice, social responsibility, and global equity.^14,15^

A particularly understudied consequence of this outflow involves its impact on future healthcare professionals—specifically, undergraduate nursing students. During their formative training years, students shift from theoretical coursework to immersive clinical practice,^16^ developing a professional identity grounded in core nursing values,^17^ ethical principles, and a sense of responsibility toward their patients and the profession itself.^18,19^ However, the widespread departure of experienced nurses introduces unique ethical complexities. On the one hand, these students observe a strained healthcare environment—overworked staff, limited mentoring, and insufficient resources—which can reinforce feelings of “moral distress”—the psychological discomfort that arises when one cannot act on perceived ethical obligations.^20^ On the other hand, the lure of professional growth and improved quality of life abroad can appear overwhelmingly attractive.^12,21^ Such conflicting pressures thrust students into dilemmas that test core ethical principles like beneficence, fidelity, and justice—particularly when the needs of the home country clash with their personal aspirations.

In Nepal’s context, there is a lack of systematic exploration into how these students reconcile these conflicting moral claims—or how witnessing the exodus of practicing nurses shapes their emerging professional identities and career ambitions. While existing research on nurse migration extensively documents push-pull factors, economic consequences, and policy-level responses,^22–25^ fewer studies probe the formative ethical perspectives of nursing students who are next in line to enter this precarious workforce. Addressing this gap is vital for both ethical theory—illuminating how moral values develop in response to real-world pressures—and practical policymaking, as strategies to mitigate migration often overlook the students’ vantage point.

This study thus aims to provide a nuanced, context-specific understanding of the ethical implications of nurse “brain drain” for undergraduate nursing students in Nepal. This research emphasizes the importance of prioritizing student perspectives—frequently marginalized within broad macroeconomic and policy-oriented dialogues. It explores how students navigate ethical dilemmas within a framework that requires localized engagement while also presenting the allure of global opportunities. Findings are expected to inform targeted approaches to nurse retention, curricular reforms in nursing ethics education, and systemic improvements within Nepal’s healthcare sector. Equally important, these insights will likely resonate with other LMICs facing a similar “brain drain,” contributing to a more global conversation about healthcare workforces’ moral integrity and sustainability. Therefore, the central research question guiding this inquiry is: What are the perceived ethical implications of nurse brain drain on undergraduate nursing students in Nepal during their formative years of training?

## Methods

### Design

This study adopted a descriptive phenomenological approach to illuminate how undergraduate nursing students in Nepal perceive and experience the ethical implications of nurse brain drain. Grounded in Husserlian philosophy,^26^ descriptive phenomenology prioritizes the epoche (bracketing) of researchers’ preconceptions, thereby striving for as unobstructed a view of participants’ lived experiences.^27,28^ Within this paradigm, the research team aimed to capture the “essence” of participants’ moral reflections by maintaining rigorous reflexive practices and honoring the meanings attributed by the students themselves.

To uphold these principles, the researchers engaged in reflexive journaling before and throughout data collection, systematically identifying any personal biases about nurse migration. This bracketing sought to prevent unintentional projection of researchers’ assumptions onto participants’ accounts. During the interviews and subsequent analysis, the team cultivated an open, inductive mindset to ensure that thematic development arose from participants’ descriptions rather than pre-existing theoretical constructs. Additionally, an imaginative variation phase allowed for the exploration of multiple interpretive angles, challenging initial impressions and fostering a deeper understanding of the phenomenon’s complexity.^29^ Integrating these key steps within a Colaizzi-style analytical structure helped maintain phenomenological rigor while also offering a clear, systematic procedure for identifying salient themes

### Population

The target population for this study consisted of undergraduate nursing students enrolled in accredited nursing programs in Nepal. The inclusion criteria were as follows: (1) registered in the third or fourth year of a Bachelor of Science in Nursing (BSc Nursing) program; (2) had completed foundational coursework and possessed clinical exposure through placements or rotations; and (3) demonstrated readiness and willingness to discuss their views on nurse migration and associated ethical dilemmas.

This focus on third- and fourth-year students ensured participants had sufficient real-world interaction with the Nepalese healthcare system—including witnessing staff shortages and resource constraints—while still being in the formative stages of professional identity development. Students in their first or second year were excluded to minimize potential gaps in direct clinical observation. Additionally, students enrolled in postgraduate programs were excluded because their more advanced stage of professional development and distinct career trajectories could yield substantially different ethical considerations about migration. To further enhance the credibility of participant selection, recruitment materials emphasized the voluntary nature of the study, mitigating potential coercion and encouraging those with genuine insights or strong interest in nurse migration to come forward.

### Setting

The study was conducted at an urban tertiary institution,^30^ situated in Bharatpur within the Bagmati province of Nepal.^31^ This institution was chosen for its prominent role in nursing education, as well as its location in a relatively urban and resource-rich region. Bagmati province is recognized for its higher concentration of healthcare facilities and comparatively more advanced infrastructure, which can expose students to a wider range of clinical scenarios, including direct experiences with the challenges that fuel nurse migration.^32^

Centering on one tertiary institution allowed for an in-depth exploration of students’ lived experiences without the logistical variances that might arise from multiple sites. Nevertheless, the specificity of the institutional environment must be acknowledged as a contextual factor shaping participants’ experiences. Nepal exhibits significant heterogeneity in healthcare infrastructure across its provinces.^33^ Students in more rural or remote areas contend with additional barriers.^34,35^ These disparities could influence their perceptions of brain drain and ethical responsibilities differently than in an urban setting. While Bagmati province provides fertile ground to study the phenomenon of outward nursing migration, follow-up investigations might compare student perspectives in other rural/remote provinces to capture how cultural, economic, and infrastructural contexts shape ethical decision-making. Such comparative work could further validate or refine the insights derived from the present study.

### Participant recruitment

This study utilized a sampling approach combining purposive^36^ and convenience^37^ methods to recruit participants who could provide rich insights into the ethical implications of nurse brain drain. The initial recruitment phase relied on convenience sampling, leveraging accessibility and participant willingness. Flyers and posters were placed at strategic locations throughout the institution. Each recruitment material clearly stated the study’s purpose and featured a QR code that linked to a secure online self-registration platform. This digital approach capitalized on the ubiquity of smartphones among the target demographic, offering a confidential and convenient way for interested individuals to volunteer.

From this initial pool of volunteers, a purposive sampling strategy was employed to select the final participants. While all volunteers met the inclusion criteria, the primary purpose was to ensure a balanced representation of students from both the third and fourth years of their program, which was deemed crucial for capturing perspectives from different stages of formative training. This approach, while grounded in convenience, was guided by the specific purpose of achieving variation^38^ in academic progression within the undergraduate nursing experience. The first author contacted each self-registered individual to verify their academic year and willingness to participate. A detailed information sheet outlining the study aims, procedures, data management plans, and their right to withdraw without penalty was provided. Initially, 18 undergraduate nursing students expressed interest through the online self-registration platform and met the eligibility criteria. From this pool, two students were invited to participate in pilot interviews, which were conducted solely to refine the interview guide. As per the informed consent agreement for the pilot phase, data from these two interviews were not included in the final analysis. The remaining 16 students were subsequently invited to participate in the main study. All 16 provided informed consent and were included in the final sample. This multi-stage approach ensured that while the initial recruitment was open and based on convenience, the final selection was purposively curated to enhance the richness and diversity of the data.

### Data Collection

Data collection took place between October 2024 and January 2025, employing in-depth, semi-structured interviews to capture the nuanced perspectives of the participants. Prior to conducting the full set of interviews, the interview guide was pilot tested with two third-year nursing students who met the eligibility criteria but were excluded from the final analysis. Feedback from this pilot phase led to minor revisions in question wording and order, thus enhancing clarity and relevance. The resulting interview guide (Table 1) reflected both a rigorous review of existing literature and the authors’ expertise in nursing ethics and migration.

**Table 1.** Interview questions.

Question number	Interview question
1	Can you describe your understanding of why many Nepali nurses choose to work abroad?
2	What are your thoughts and feelings about the number of nurses leaving Nepal to work in other countries?
3	Have you personally considered working as a nurse outside of Nepal? What factors influence your thinking on this?
4	Have you felt any pressure (from family, friends, or society) to consider working abroad? If so, can you describe that pressure?
5	Have you encountered any situations during your training that have made you question the ethics of nurses leaving Nepal? Can you describe them?
6	How do you feel the migration of nurses impacts the quality of healthcare in Nepal?
7	How has observing the migration of nurses affected your own view of the nursing profession in Nepal?
8	What do you think are the biggest challenges facing the Nepalese healthcare system in relation to nurse staffing?
9	What, if anything, do you think could be done to encourage more nurses to stay and work in Nepal?
10	If you could speak directly to policymakers about this issue, what would you want them to know?

All formal interviews were conducted face-to-face by the first author in a quiet, private room within the campus library, a location chosen to promote comfort and confidentiality. Each session, lasting between 45 and 75 min, began with general, open-ended questions designed to encourage candid reflection, followed by more focused probes. Participants were reminded that there were no right or wrong answers, reinforcing the study’s emphasis on understanding their lived experiences rather than eliciting predetermined responses. Interviews were audio-recorded, and informed consent was reconfirmed at the start of each session.

### Data analysis

Consistent with descriptive phenomenology, data analysis followed Colaizzi’s method,^39^ which provided a structured yet flexible pathway to identifying and elaborating core themes that reflect participants’ lived experiences. First, the first author (AG) transcribed all audio-recorded interviews verbatim. The first author is a policy-oriented researcher with extensive experience as a nurse educator and clinician in diverse Nepalese healthcare settings. This background, which includes involvement in health policy research and committees, provided a strong foundation for conducting in-depth qualitative inquiry. This transcription process enabled thorough immersion in the data. Each transcript was read multiple times to foster familiarization, during which researchers recorded any immediate impressions in their reflexive journals.

Next, the research team extracted significant statements, phrases, or segments that directly illuminated participants’ ethical reasoning about nurse migration. Each statement was then assigned a formulated meaning, reflecting the essential insight or concept underlying the participant’s words. Through iterative comparison and discussion, these formulated meanings were clustered into sub-themes that captured recurring patterns and distinct nuances in the data.

**Figure 1.**
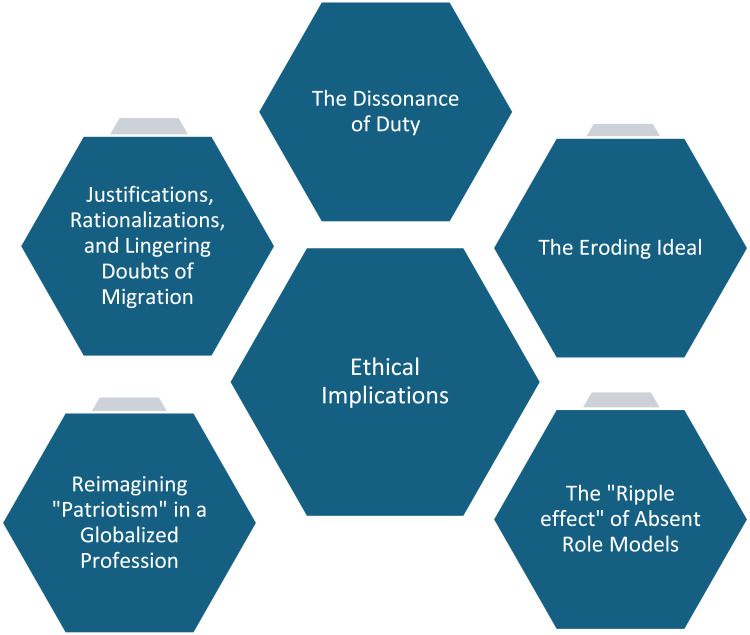
The ethical implications of nurse brain drain on Nepalese undergraduate nursing students.

Aligned with phenomenological reduction, the researchers carefully revisited the transcripts to confirm that these sub-themes remained true to participants’ words, avoiding any overlay of external frameworks. In keeping with the principle of imaginative variation, the team tested alternative interpretations to ensure that each sub-theme was robust and accounted for the range of experiences described. Following this scrutinizing process, the sub-themes were distilled into overarching themes that represented the core ethical dilemmas and moral perspectives articulated by the students (Figure 1).

Throughout the analysis, the researchers worked both independently and collaboratively to code and categorize the data, with any interpretive discrepancies resolved via sustained dialogue and re-examination of the primary material. This iterative, consensus-building process not only strengthened the credibility of the findings but also preserved the authenticity of the participants’ voices. Once it became evident that no further novel insights were emerging, a point commonly referred to as thematic saturation^40,41^—the researchers concluded that the essential phenomena had been thoroughly represented.^42^

By adhering to Colaizzi’s descriptive phenomenological method, the study balanced phenomenological depth with a transparent analytic structure. The resulting themes thus emanate directly from the students’ experiences, free to the greatest extent possible from researcher preconceptions. This unified approach underscores the study’s commitment to capturing the richness and complexity of nursing students’ ethical landscapes surrounding brain drain, while remaining faithful to the methodological rigors of descriptive phenomenology (Table 2).

**Table 2.** Illustrative data analysis using Colaizzi’s method.

Significant statement (illustrative quote)	Formulated meaning	Meaning cluster	Sub-theme	Overarching theme
“My father always says, ‘our country needs you. Who will take care of our people if all the nurses leave?’ … it feels like a constant battle inside.” (P1)	Feeling torn between familial/societal expectations to serve the nation and personal aspirations for a better life.	Familial / Social pressures	Family expectations and duty	The dissonance of duty
“I believe I can do more good, even for Nepal, by gaining experience and resources abroad. Maybe I can even come back 1 day, better equipped to make a difference.” (P2)	Perceiving migration as a strategy to gain skills, potentially benefiting the home country in the long run despite short-term absence.	Future-oriented perspective	Long-term benefit vs. Short-Term absence	The dissonance of duty
“In my clinical rotations, I see the exhaustion on the faces of the senior nurses… it makes me wonder, is this what I’m signing up for? Is this all nursing is in Nepal – a constant struggle against impossible odds?” (P5)	Observing severe work stress in practicing nurses leads to doubts about remaining in a system perceived as undervalued and overburdened.	Systemic strain	Burnout and overwork	The eroding ideal
“We learn about patient-centered care… but in the hospitals, it feels like everything is rushed… it makes you wonder if the ideals we’re taught are even achievable here.” (P6)	Experiencing a disconnect between nursing ideals taught in school and the reality of overloaded wards, resulting in compromised care delivery.	Discrepancy between theory and practice	Compromised care standards	The eroding ideal
“I tell myself that I can do more good for Nepal by sending money back… Isn’t that a kind of service, too? It’s not the same as being here, I know, but it’s something.” (P9)	Justifying migration through remittances as an indirect contribution to the home country, yet acknowledging it is not equivalent to hands-on care.	Economic rationalization	Financial support vs. Physical presence	Justifications, rationalizations, and lingering doubts of migration
“Sometimes, I lie awake at night, thinking about the patients I’ve cared for… Am I making the right choice? Am I letting them down? … It’s a constant struggle. I don’t think there’s an easy answer.” (P11)	Expressing guilt and uncertainty about choosing personal advancement over patient needs, reflecting deep moral ambivalence.	Ethical ambivalence	Guilt and uncertainty	Justifications, rationalizations, and lingering doubts of migration
“In our classes, we learn about leadership… but in the hospital, it’s hard to find those examples… it feels like there’s a gap between what we’re taught and what we see.” (P13)	Lamenting the lack of mentors who embody leadership qualities, leading to a perceived disconnect between educational ideals and clinical practice.	Mentorship deficiencies	Disconnect between theory and practice	The ‘ripple effect’ of absent role models
“I sometimes wonder if the nurses who leave had mentors when they were students […] maybe it’s a cycle, and we’re just caught in it.” (P16)	Suspecting a cyclical dynamic where the absence of mentors contributes to more nurses leaving, perpetuating long-term workforce gaps.	Intergenerational impact	Long-term consequences of brain drain	The “ripple effect” of absent role models
“Is it truly ‘unpatriotic’ to want a better life? … It’s not about escaping; it’s about expanding our horizons and, ultimately, contributing in a different way.” (P17)	Challenging traditional notions of loyalty to one’s country, reframing migration as an extension of global citizenship and future contribution.	Reimagining patriotism	Challenging traditional notions of loyalty	Reimagining “patriotism” in a globalized profession
“We talk about ‘brain drain,’ but what about ‘brain gain’?… maybe the problem isn’t migration itself, but the lack of a system that encourages and supports the return of skilled professionals.” (P18)	Proposing an alternative viewpoint that envisions migration as potentially beneficial if returning professionals are welcomed and incentivized.	Structural responsibility	Reframing migration as an opportunity	Reimagining “patriotism” in a globalized profession

### Rigor and reflexivity

The credibility, transferability, dependability, and confirmability of this qualitative study were ensured through multiple, interrelated strategies.^43^ In terms of credibility, or the trustworthiness of the data and interpretations, the researchers engaged in prolonged immersion by repeatedly examining the transcripts, conducting iterative questioning during interviews, and comparing coded segments independently before arriving at a consensus. Member checking further strengthened credibility: a concise summary of preliminary findings was shared with each participant, inviting them to confirm or challenge the researchers’ interpretations. Their feedback, which offered clarifications and occasional new insights, was incorporated into the final analysis, ensuring the findings faithfully reflected the participants’ lived experiences.

To promote transferability, the study presents thick, detailed descriptions of the setting, participant demographics, and research processes. While the findings emerge from a Nepalese context, the comprehensive methodological account enables readers in other LMICs to determine whether the insights might resonate within their own healthcare environments. Regarding dependability, the authors maintained an audit trail of decisions made during data collection and analysis. This documentation covered details such as adjustments to interview questions and the rationale for coding categories, thus permitting external scrutiny of how the study evolved. The semi-structured interview guide, coupled with an explicitly collaborative approach to data analysis, further reinforced dependability by providing a systematic framework that minimized variations in data handling.

Confirmability was addressed by explicitly minimizing researcher bias throughout all stages. The rigorous coding process included independent coding by both authors, followed by joint reviews of any discrepancies. Such consistent cross-examination ensured that the emerging themes stemmed from the data rather than from any individual’s predispositions. Additionally, the authors had no prior relationship with any of the participants, which helped maintain a neutral stance toward the data.

Reflexivity, a critical component of qualitative research,^44^ was central to this study. The research team acknowledged their own positionalities and potential biases throughout the research process. The first author, a migrant nurse and academic working overseas, brought an “insider-outsider” perspective,^45^ possessing an understanding of the motivations and challenges of migration while also maintaining a degree of distance from the immediate context of Nepalese nursing students. The second author (MSN), a highly experienced nursing researcher and academic deeply embedded in the Nepalese healthcare system, provided a complementary “insider” perspective grounded in years of experience and local knowledge. This combination of perspectives was invaluable in navigating the complexities of the research topic. The research team’s differing backgrounds and potential biases were actively discussed and critically examined throughout the data collection and analysis process. This reflexive dialogue served as a check on individual interpretations, ensuring that any single researcher’s preconceived notions did not unduly influence the findings. The explicit acknowledgment of researcher positionality enhances the transparency and trustworthiness of the study, allowing readers to critically evaluate the interpretations presented.

### Ethical considerations

Ethical approval for this study was obtained from the Nepal Health Research Council (NHRC-277/2024) and the internal review board of the participating tertiary institution. Written informed consent was secured from all participants prior to data collection. Each participant received a comprehensive information sheet and was explicitly advised of their right to confidentiality, anonymity, and the ability to withdraw from the study at any time without penalty.

## Results

### Participants characteristics

All 16 participants were female, a demographic composition reflecting the broader gender distribution within Nepal’s nursing profession, which remains overwhelmingly female-dominated.^7^ Their ages ranged from 21 to 26. The participants were divided between third-year (*n* = 6) and fourth-year (*n* = 10) cohorts, ensuring a breadth of clinical exposure and developmental stages. Students in their third year had already completed foundational coursework and were undertaking their required clinical rotations, while fourth-year students had more extensive hands-on experience and were approaching the transition to professional practice. Consistent with national educational pathways, all participants had entered the 4-year Bachelor of Science in Nursing (BSc Nursing) program^46^ program after completing Year 12 secondary education.^3^ The majority of students reported nursing as their first-choice career, a notable subset indicated that they had initially considered alternative professions. This mixture of career intentions and academic seniority enriches the qualitative dataset, offering insights into how varying levels of clinical engagement and personal aspirations may shape the ethical considerations around nurse migration. Table 3 summarizes the participants’ socio-demographic characteristics, including their academic year, age, and whether nursing was their first-choice profession.

**Table 3.** Participants’ socio-demographic characteristics.

Participant	Gender	Previous education	Year in nursing program	Nursing as first choice
P1	Female	High school	4th year	Yes
P2	Female	High school	4th year	Yes
P3	Female	High school	3rd year	Yes
P4	Female	High school	4th year	Yes
P5	Female	High school	3rd year	No
P6	Female	High school	4th year	Yes
P7	Female	High school	4th year	No
P8	Female	High school	3rd year	Yes
P9	Female	High school	4th year	Yes
P10	Female	High school	3rd year	No
P11	Female	High school	4th year	Yes
P12	Female	High school	3rd year	No
P13	Female	High school	4th year	Yes
P14	Female	High school	4th year	No
P15	Female	High school	3rd year	Yes
P16	Female	High school	4th year	Yes

## Findings

### Theme 1: The dissonance of duty

Participants repeatedly voiced a deep-seated tension between what they perceived as their moral responsibility to serve Nepal’s healthcare needs and their compelling desire for professional advancement, improved working conditions, and personal fulfillment abroad. This tension was neither trivial nor singular; rather, it reflected a multifaceted ethical landscape influenced by familial pressure, socioeconomic realities, and the perceived undervaluation of the nursing profession within Nepal.

“My father always says, ‘Our country needs you. Who will take care of our people if all the nurses leave?’ I see the desperation in his eyes. My father has worked hard to put me through university […] And he’s right, the hospitals are so short-staffed. But then I see my seniors, working double shifts, exhausted, barely making enough to get by. I want to help my family, to have a better life, to learn new skills. Is that selfish? I don’t know anymore[...] It feels like a constant battle inside.” (P1)

This narrative underscores the intimate, familial dimension of the dilemma, where students must reconcile parental expectations and nationalistic appeals with the realities of low compensation and inadequate institutional support. Their struggle transcends mere abstraction; it is lived out daily, rooted in tangible sacrifices made by both their parents and themselves.

“Of course, I feel a responsibility to Nepal. We all do. We wouldn’t be in nursing if we didn’t care about people. But I also have a responsibility to myself and to my future. I’ve seen nurses with years of experience struggling financially […]. I don’t want that for myself. I believe I can do more good for Nepal by gaining experience and resources abroad. Maybe I can even come back one day, better equipped to make a difference.” (P2)

Here, the push-pull forces of migration become evident. The student seeks to reconcile her sense of national duty with the potential for “brain circulation,” wherein time spent abroad could ultimately benefit Nepal if newly acquired skills and resources are later repatriated.^47^ Yet this explanation also suggests rationalization—a means to alleviate the moral burden of leaving a strained system.

“It’s not just about the money, though that’s obviously a huge part of it. It’s about respect. Here, nurses are often treated as assistants, not as professionals. Abroad, I hear they have more autonomy, more opportunities to specialize, to really use their skills. I want to be valued for what I do. I want to feel like I’m making a real impact. Is that too much to ask?” (P15)

The conflict in this account highlights professional respect as a critical driver. While economic considerations underlie many migration decisions, students repeatedly stressed the importance of being recognized as skilled healthcare providers rather than peripheral aides. Feeling undervalued can intensify the internal strife between serving the nation and pursuing an environment that honors their expertise.

“I went on a placement to a rural health post last semester. It broke my heart. So few resources, so many patients in need. I felt this overwhelming urge to stay, to help. But then I talked to the nurses there. They were burned out, cynical [...] They told me to get out while I could, that nothing would change.” (P12)

This reflection captures the emotional and moral jeopardy inherent in remaining within a system perceived to be unreceptive to change. The student initially experiences an altruistic desire to serve in a vulnerable setting but encounters burnt-out practitioners who warn her against entrapment in an under-resourced environment. The possibility of disillusionment emerges as a strong counterweight, illustrating the fragile balance between devoting oneself to a national cause and preserving one’s professional ideals.

### Theme 2: The eroding ideal

Participants consistently reported an unsettling dissonance between the ideals they had internalized during their nursing education and the stark realities observed within Nepal’s healthcare settings. Their accounts underscored not only the systemic strain caused by understaffing and limited resources but also the corrosive effect such conditions exert on their sense of professional worth, pride, and optimism about the future of nursing in Nepal.

“In our classes, we learn about leadership, about advocating for patients, about being a role model. But in the hospital, it’s hard to find those examples. Patients are told to go and get their own medical supplies with this pay-as-you-go system […] Yet the constitution guarantees free healthcare for all. It feels like there’s a gap between what we’re taught and what we see. How am I learning the core values of what makes a good nurse?” (P13)

This reflection exposes a theory-practice divide. Students find themselves in a paradox: they must internalize ideals like advocacy and leadership, yet the scarcity of resources undermines their ability to translate classroom philosophies into clinical wisdom.

“We learn about patient-centered care, about holistic approaches, about the importance of empathy. But in the hospitals, it feels like everything is rushed, everything is about just getting through the day. There’s no time for the ‘niceties,’ for the things that make nursing more than just a job. It makes you wonder if the ideals we’re taught are even achievable here.” (P6)

By contrasting theoretical ideals (holistic care, empathy, patient advocacy) with the pragmatic realities of an overburdened system, this participant sheds light on the gap that can breed disillusionment. While undergraduate curricula strive to inculcate a high standard of practice, the stressful circumstances on the ground appear to erode students’ belief in their profession’s transformative potential. This rift between expectation and practice risks normalizing a culture of minimalism, where students grow accustomed to merely meeting the basic needs of patients rather than fully embodying compassionate, holistic nursing.

“My aunt is a nurse in the UK; she was recruited through the Nepal-UK agreement. She describes – the support, the resources, and the professional respect as quite different compared to Nepal […]. Then I look at our hospitals here [...] it's hard not to feel like we're fighting a losing battle. It makes you question everything – the system, your own future, whether things will ever really change.” (P7)

For many participants, international comparisons intensify feelings of disempowerment. Encountering stories of nurses who thrive abroad, equipped with comprehensive resources and professional autonomy, underscores their perceived devaluation within Nepal. The mismatch between these contrasting realities not only prompts students to consider migration themselves but also instills doubt about whether systematic reforms are feasible in their home country.

### Theme 3: Justifications, rationalizations, and lingering doubts of migration

Participants described a deeply personal, often agonizing internal debate as they weighed the potential benefits of working abroad against the ethical implications of leaving Nepal’s already overburdened healthcare system. This “moral calculus”^48^ was grounded in both rational justifications—such as remittances, skill acquisition, and long-term national benefit—and emotional undercurrents of guilt, duty, and uncertainty. Family obligations, peer pressures, and broader societal narratives further complicate these decision-making processes, underscoring the complexity of nurse migration in a resource-constrained environment.

“I tell myself that I can do more good for Nepal by sending money back. My family depends on me. If I stay here, we’ll all struggle. If I go abroad, I can support them, educate my siblings, maybe even help build a better house. Isn’t that a kind of service, too? It’s not the same as being here, I know, but it’s something.” (P10)

For this participant, economic remittances emerged as a central rationale for contemplating migration, framed as a pragmatic way to fulfill familial and national obligations. Yet the hesitation in her tone—“It’s not the same as being here”—reveals an acute awareness that financial support cannot replace the direct care and presence she would provide if she remained in Nepal. Consequently, her reasoning reflects a delicate blend of self-justification and unresolved ethical unease, illustrating the ambiguity inherent in seeking both personal security and collective well-being.

“I’ve heard some people say we’re being selfish, that we’re abandoning our country. But I don’t see it that way. I’m not running away; I’m seeking opportunities to grow, to become a better nurse. I believe I can bring those skills back someday. I want to come back. But I need to build a foundation first. It is not like I will forget Nepal.” (P4)

This account captures a temporal dimension to the moral calculus. By construing migration as a temporary stage, the student reframes the act of leaving as an investment in future capacity-building for Nepal. Such narratives of “brain circulation” both mitigate accusations of selfishness and offer hopeful visions of eventual return. Yet whether students who depart truly bring new competencies back or remain permanently abroad remains uncertain, revealing a tension between idealistic aspirations and more pragmatic outcomes.

“Sometimes, I lie awake at night, thinking about the patients I’ve cared for, the people who need help. I feel this […] pull, this guilt. Am I making the right choice? Am I letting them down? But then I think about my own future, my own well-being. It’s a constant struggle. I don’t think there’s an easy answer.” (P11)

In this portrayal of emotional turmoil, the student acknowledges an ethical conflict that is neither neatly addressed by financial rationales nor by promises of future return. The gnawing sense of guilt reflects her recognition that, despite her personal aspirations, the decision to leave may exacerbate existing staffing deficits and negatively affect patient care. This ethical ambivalence remains unresolved, underscoring that migration choices are not solely driven by economics but also by deep moral introspection and an awareness of collective vulnerability.

“Many of my seniors plan to, or have already, left. I even have a friend in nursing school with me, and her sole goal is to go abroad. They have done all of the calculations. They are all set. It is hard to avoid their discussions, and I am slowly being pulled into it. I know nurses are desperately needed in Nepal; I see the impact. However, the pull is so strong […] Everyone keeps telling us there is no future here.” (P3)

Here, the social dimension of the moral calculus comes into sharp relief. Observing peers and senior nurses systematically opting for emigration casts staying in Nepal as an increasingly countercultural choice. The near-constant dialogue about leaving—and the explicit messaging that “there is no future” in the local healthcare system—magnifies the internal struggle, reinforcing the notion that departing might be the only viable avenue for professional advancement and financial security.

### Theme 4: The “ripple effect” of absent role models

Participants articulated a less overt, yet profoundly influential consequence of nurse migration: the diminishing pool of seasoned professionals able to mentor and guide emerging nurses. This theme underscores how the shortage of experienced role models reverberates through students’ educational experiences and shapes their evolving sense of professional identity, ethical judgment, and communal ties within the field.

“In my clinical rotations, I see the exhaustion on the faces of the senior nurses. They’re constantly running, always behind. They barely have time to teach us, let alone provide the kind of care they want to give. It makes me wonder, is this what I’m signing up for? Is this all nursing is in Nepal—a constant struggle against impossible odds?” (P8)

In this reflection, the physical and emotional exhaustion of senior nurses becomes a stark counterpoint to the participant’s formative ideals. Rather than viewing nursing as a rewarding vocation, she perceives it as a relentless contest against resource limitations and insufficient staffing.

“I wish I had a mentor, someone I could really talk to about the challenges, the ethical dilemmas. Someone who could show me how to navigate the system, how to stay true to my values even when it’s hard[…] But most of the nurses I admire have already left. It is a bit lonely; I am not going to lie.” (P16)

Here, the participant’s yearning for mentorship highlights the emotional toll exacted by nurse shortages and high turnover rates. In addition to imparting clinical knowledge, effective mentors can guide novices through ethically ambiguous situations and reinforce their resilience.

“We rely a lot on each other, on our classmates. We share our experiences, our frustrations, our fears. It’s helpful, but it’s not the same as having someone who’s been through it all, someone who can offer guidance from a place of experience. We are all just trying to make it from one clinical placement to the next.” (P15)

This student explains that horizontal support among classmates fills some gaps by fostering solidarity and emotional coping. Nevertheless, peers are similarly inexperienced, lacking the seasoned perspectives and strategic insights that long-tenured nurses can provide. Without consistent mentoring relationships, students risk developing fragmented professional identities founded on survival tactics rather than aspirational leadership models.

“I sometimes wonder if the nurses who leave had mentors when they were students. Did they have someone to help them navigate the challenges? Maybe if they had, they wouldn’t have left. Maybe it’s a cycle, and we’re just caught in it.” (P13)

This quote powerfully encapsulates the intergenerational implications of nurse migration. The student posits a cyclical dynamic, hypothesizing that those who depart may have done so due to inadequate support and guidance. As migration persists, it depletes the stock of experienced nurses who might otherwise serve as mentors, thus perpetuating the likelihood of future cohorts similarly losing a sense of hope and professional anchor.

### Theme 5: Reimagining “patriotism” in a globalized profession

Participants frequently contested the assumption that remaining physically in Nepal is the only meaningful way to demonstrate patriotism, instead positing that a globalized nursing profession allows for diverse modes of national service. Their reflections reveal a shift from viewing migration as a purely individualistic endeavor toward perceiving it as part of a broader, interconnected strategy for national development. In so doing, they redefine “patriotism” to encompass not only immediate bedside contributions at home but also the dissemination of skills and international advocacy on behalf of Nepal.

“Is it truly ‘unpatriotic’ to want a better life? We’re not abandoning Nepal; we’re becoming part of a global Nepali community. We can be ambassadors, sharing our culture, raising awareness about our country’s needs. And frankly, the skills and experience we gain abroad will make us more valuable if we do return. It’s not about escaping; it’s about expanding our horizons and, ultimately, contributing in a different way.” (P15)

Here, the notion of a “global Nepali community” challenges traditional interpretations of loyalty, suggesting that migration can expand rather than diminish one’s ability to serve Nepal. By characterizing transnational nurses as “ambassadors,” the participant underscores a vision of healthcare work as border-spanning, whereby accrued expertise abroad may eventually reinforce the local system. This reframing highlights how knowledge transfer and resource sharing, when leveraged effectively, could function as a form of extended national commitment rather than a betrayal of it.

“I might work here for a few years after graduating, to gain some experience and ‘give back’ to the system. But honestly, my long-term plan is to go abroad. I think I can be a better advocate for Nepal from the outside. I can connect with international organizations, raise awareness about the challenges we face, maybe even help facilitate collaborations. It’s a different kind of patriotism, maybe, but it feels more realistic.” (P1)

In this reflection, the student envisions a “balancing act” between short-term commitments at home and longer-term contributions from abroad, grounded in the belief that operating internationally could amplify her capacity to effect change. Whether this approach yields tangible benefits for Nepal’s health system depends on diverse factors—including policy frameworks that facilitate return or encourage remote collaboration. Nonetheless, her testimony reinforces the idea that patriotism transcends mere physical presence.

“We talk about ‘brain drain,’ but what about ‘brain gain’? If we go abroad, learn new skills, and then choose to come back—not because we have to, but because we want to—wouldn’t that be a powerful contribution? Maybe the problem isn’t migration itself, but the lack of a system that encourages and supports the return of skilled professionals.” (P11)

Participants contend that mere moral exhortations aimed at nursing retention are insufficient to address the complexities of the issue. They advocate for a perspective centered on the potential for “brain gain,” which emphasizes the importance of recognizing systemic factors. This approach shifts the responsibility from individual decision-making to the necessity of enacting broad policy interventions designed to cultivate an environment that is supportive and conducive to the reintegration of healthcare professionals.

## Discussion

This study’s findings reveal a complex and multifaceted ethical landscape navigated by Nepalese nursing students as they contemplate their professional futures amid a substantial healthcare worker migration. The data uncovers a profound tension between personal and professional values, the realities of a strained healthcare system, and the allure of opportunities abroad. Students grapple with conflicting obligations to their country, their families, and their own aspirations, engaging in intricate moral reasoning to justify their choices. The observation of systemic challenges within Nepal, coupled with the absence of experienced mentors, impacts their immediate learning environment and their long-term perceptions of the nursing profession’s overall value and viability within their nation. Furthermore, traditional notions of national service undergo re-evaluation as students explore pathways for contributing to Nepal’s well-being in a globalized context. These intertwined factors demand an understanding of the ethical implications of brain drain, moving beyond simplistic narratives to embrace the complexities of individual decision-making and the broader systemic forces at play.

The narratives surrounding “The Dissonance of Duty” reveal a profound ethical tension at the core of career decisions for Nepalese nursing students. This is not a simple choice between staying and leaving; it is a deeply moral struggle, interwoven with familial obligations, societal expectations, and the inherent altruism of nursing.^3,49^ Participants, like P1, described the significant weight of family expectations, reflecting the cultural emphasis on filial piety and contributing to the family’s economic well-being.^50^ This pressure clashes with the harsh realities of the Nepalese healthcare system—low wages, limited career advancement, and demanding working conditions^3,51^—creating a powerful incentive to seek opportunities abroad. This conflicts with the core ethical principle of beneficence, the duty to act in the best interests of others.^52^ Students are torn between their desire to improve the well-being of their families and their potential to contribute to Nepal’s health system. This internal conflict often results in moral residue^53^—a lingering sense of unease or guilt, even after a decision is made, because the unchosen option still holds moral weight. The Nepal-UK memorandum of understanding (MoU) on nurse recruitment,^13^ while presenting individual opportunities, further complicates this ethical landscape by raising questions of global justice and the potential to worsen existing healthcare disparities. Ultimately, these students confront a personal yet universally significant ethical dilemma: balancing competing moral obligations in a world of profound systemic inequality. Their struggle highlights the broader global challenge of healthcare worker migration, demanding solutions that address both individual aspirations and collective well-being.

The psychological and ethical ramifications of witnessing a healthcare system under stress were clearly reflected in the participants’ accounts. The poignant testimonies describing exhausted nurses and compromised patient care paint a stark picture of a profession struggling to uphold its core values.^54^ This dissonance between the taught ideals of nursing—empathy, holistic care, patient advocacy—and the observed realities of understaffing and resource scarcity^12^ creates a breeding ground for moral distress. From a pedagogical perspective, this gap presents a significant challenge. Nursing education often emphasizes ethical principles and best practices^55^ that, while aspirational, may seem unattainable in the current Nepalese context. This can lead to cynicism and a questioning of the profession’s worth, as voiced by P7, who compared the Nepalese system unfavorably to that of the UK. The phenomenon of disillusionment transcends mere individual disappointment and could have profound implications for the future of the nursing profession in Nepal. A disenchanted workforce is less inclined to remain within the country, thereby exacerbating the existing brain drain crisis.^1^ This creates a vicious cycle: systemic strain leads to devaluation of the profession, which in turn propels more emigration. Observing this dysfunction can induce learned helplessness, wherein students perceive themselves as incapable of effecting meaningful change, thereby undermining professional identity.

“Justifications, Rationalizations, and Lingering Doubts of Migration” exposes the intricate internal negotiations shaping Nepalese nursing students’ contemplation of working abroad. Instead of merely conducting a straightforward evaluation of advantages and disadvantages, this process necessitates intricate moral deliberation, wherein students must navigate and weigh ethical principles and responsibilities. Participant justifications centering on remittances serve as a common rationalization—viewing migration as a route to fulfill familial obligations and indirectly support Nepal’s well-being.^56,57^ This approach aligns with a utilitarian ethical framework, seeking the “greatest good,”^58^ even if it involves personal sacrifice. However, as students’ candid disclosures of guilt and doubt show, this calculus is rarely straightforward. The philosophical concept of moral distress surfaces again, manifesting as conflict between personal advancement and the knowledge of deserting a system in need. Students’ assertions that returning later with refined skills will ultimately benefit Nepal mirror “moral licensing,”^59^ implying that an intention to perform future beneficial actions can justify ethically ambiguous decisions now. This underscores a key tension: Are such narratives earnest resolutions, or do they function as coping mechanisms to mitigate the cognitive dissonance inherent in decisions fraught with ethical weight? Moreover, the influence of social narratives, illustrated by P12’s observation of how peers normalize migration, highlights the role of social psychology in shaping individual choices. The moral calculus is far from solitary; it is deeply embedded in a context where leaving is increasingly deemed a logical route to professional success.

Migration, frequently regarded as an avenue to career growth, illuminates a significant yet frequently overlooked consequence of brain drain: the weakening of intergenerational knowledge transfer, alongside the degradation of values and professional identity within Nepal’s nursing profession. P13’s observations of a gap between classroom ideals and the reality of overworked senior nurses underscore the shortage of accessible role models who can embody those values.^60^ This absence extends beyond logistical concerns, substantially influencing the socialization of novice nurses. Forming a professional identity involves internalizing the ethos, norms, and conduct emblematic of nursing.^17,61^ Mentorship is a central mechanism for this process,^62^ offering students a tangible illustration of what nursing means within Nepal’s specific milieu. The dearth of mentors, as noted by P14, leaves students feeling “lonely” and deprived of the guidance needed to negotiate both ethical dilemmas and day-to-day professional challenges. This aligns with social learning theory, which emphasizes the importance of observation and imitation in acquiring new behaviors and attitudes.^63^ While peer support can mitigate immediate stress, it cannot match the insight mentors offer. Moreover, P16’s reflections on a potential “cycle of migration,” wherein the lack of mentors perpetuates further departures, hint at the long-term, systemic consequences of such attrition. This predicament transcends individual isolation, portending a broader erosion of the Nepalese nursing profession’s foundational knowledge, ethical grounding, and collective identity with each exit.

The commitment voiced by these participants extends beyond national boundaries, signifying a paradigm shift in how Nepalese nursing students understand their link to their homeland. The conventional association of patriotism with physical residence is being actively questioned,^64^ replaced by a more nuanced appreciation of contribution in an interconnected world. P17’s assertion that migration equates to “expanding horizons” rather than “escaping” speaks to a cosmopolitan ethos, where national identity is not confined by physical space.^65^ This viewpoint resonates with the increasingly borderless nature of the healthcare workforce, where knowledge and personnel traverse continents.^66,67^ The concept of “brain circulation,”^68^ rather than the purely negative “brain drain,” holds particular significance here. P18’s direct question—“what about ‘brain gain’?”—challenges dominant narratives. Rather than regarding emigrating nurses as lost assets, participants conceive of them as potential returns on investment, whose overseas experience might be harnessed to strengthen Nepal’s nursing workforce. However, in the present context, the nursing shortage remains acute: with a nurse-to-population ratio of 3.4 per 1000^69^ Nepal has fewer than 115,900 registered nurses for over 30 million people,^70^ underlining the severity of the issue. The narratives presented suggest that, in contemporary contexts, particularly within globally mobile professions such as nursing, the concept of “patriotism” is undergoing a significant transformation. It appears to be shifting from a traditional focus on geographical allegiance to an increased emphasis on the nature and magnitude of one’s contributions to national welfare. This perspective highlights that the impact of individuals on their home country’s well-being may transcend physical location, thereby redefining the parameters of national loyalty and engagement in a globalized world.

### Limitations and future research

While this study provides valuable insights, it is subject to certain limitations. First, as the research was conducted at a single medical college, and given its qualitative nature, the findings are not generalizable to the broader population of nursing students across Nepal. Furthermore, the recruitment method relied on voluntary participation. As such, students with a particular interest or strong feelings about nurse migration may have been more inclined to volunteer, which raises the potential for selection bias. We acknowledge that those who opted into the study might have held more pronounced views on ethical dilemmas surrounding brain drain than their peers who did not respond. Nonetheless, the process was intentionally structured to uphold participant autonomy and ensure that those who participated did so voluntarily and with full awareness of the study’s implications. Additionally, the sample comprised undergraduate students; hence, the perspectives of fully practicing nurses may diverge. Finally, the rapidly shifting global healthcare environment, including emergent bilateral agreements or policy changes, could alter student views over time, thus shaping the temporal validity of the results.

Future inquiries should address these constraints through longitudinal research that traces the evolution of students’ ethical reasoning and career trajectories, thereby illuminating how these perspectives mature under real-world pressures. Quantitative or mixed-methods designs incorporating larger, representative samples would afford the statistical power to confirm or refine these qualitative insights. Comparative studies across multiple low- and middle-income countries (LMICs) with significant nurse migration would further deepen understanding of the social, cultural, and economic factors underpinning students’ moral decision-making. Research focused on interventions—such as mentorship support or educational programs that mitigate moral distress—could yield evidence-based strategies to encourage nurse retention. Lastly, investigating policymakers’ and healthcare administrators’ views in conjunction with student perspectives may uncover the systemic levers needed to develop ethical, impactful policy solutions to nurse migration.

### Conclusions and recommendations

The findings of this study suggest a multifaceted approach to address the ethical challenges posed by nurse migration in Nepal. First, curricular reform in nursing education is crucial. Ethical instruction should extend beyond theoretical principles, encompassing real-world dilemmas associated with migration and integrating reflective exercises, case studies, and debates on professional obligations. Inviting both local and internationally practicing Nepali nurses to speak with students could further bridge the gap between academic theory and global realities.

Second, a robust mentorship program is crucial. By pairing students with experienced nurses locally and, where feasible, establishing virtual connections with Nepali nurses working abroad, future nurses can benefit from a wider network of professional guidance. Such mentorship fosters resilience, refines clinical competencies, and nurtures the ethical grounding vital for informed career decisions.

Third, tackling systemic issues within Nepal’s healthcare sector is essential. Improved working conditions, fair compensation, and more accessible professional development opportunities can collectively enhance the attractiveness of local practice, helping to curb the persistent outflow of skilled nurses. Finally, policy-level interventions should focus on ethical international recruitment. In partnership with professional associations such as the International Council of Nurses (ICN), the Nepalese government can negotiate bilateral agreements that champion “brain circulation” instead of one-way migration. Such accords might involve skill-transfer mechanisms, structured return incentives, and financial or logistical support for Nepali nurses practicing overseas.

Building upon the insights from this study, follow-up investigations might compare student perspectives in other rural/remote provinces to capture how cultural, economic, and infrastructural contexts shape ethical decision-making. Such comparative work could further validate or refine the insights derived from the present study.

In conclusion, this study reveals that Nepalese nursing students are not merely observers of brain drain; they are active participants in a complex ethical drama. They grapple with conflicting loyalties, navigate systemic pressures, and ultimately redefine what it means to serve their country in a globalized world. Their experiences underscore the urgent need to create a healthcare environment in Nepal that values and supports its nurses, fostering a sense of professional fulfillment alongside national commitment. The enduring question is not whether these students will choose to leave but whether Nepal will give them compelling reasons to stay—a question that speaks directly to the future of the nation's healthcare.

## Data Availability

The data that support the findings of this study are available on request from the corresponding author. The data is not publicly available due to privacy or ethical restrictions.*
